# Implementation resources to support teachers’ use of behavioral classroom interventions: protocol of a randomized pilot trial

**DOI:** 10.1186/s40814-023-01381-4

**Published:** 2023-08-25

**Authors:** Gwendolyn M. Lawson, Julie Sarno Owens, David S. Mandell, Samantha Tavlin, Steven Rufe, Aaron R. Lyon, Ricardo Eiraldi, Thomas J. Power

**Affiliations:** 1 Children’s Hospital of Philadelphia, Philadelphia, USA; 2 Department of Psychiatry, Perelman School of Medicine, University of Pennsylvania, Philadelphia, USA; 3 Ohio University, Athens, USA; 4 Rufe Educational Consulting, LLC, Schwenksville, PA USA; 5 Department of Psychiatry and Behavioral Sciences, University of Washington, Seattle, USA; 6 Department of Pediatrics, Perelman School of Medicine, University of Pennsylvania, Philadelphia, USA

**Keywords:** Behavioral classroom interventions, ADHD, PBIS

## Abstract

**Background:**

Teacher-delivered behavioral classroom management interventions are effective for students with or at-risk for attention-deficit/hyperactivity disorder (ADHD) or other disruptive behavior challenges, but they can be difficult for teachers to use in the classroom. In this study, we will pilot test a package of implementation strategies to support teachers in using behavioral classroom interventions for students with ADHD symptoms.

**Methods:**

We will use a 2-group, randomized controlled trial to compare outcomes for teachers who receive Positive Behavior Management Implementation Resources (PBMIR), a theory and data-driven implementation resource package designed to increase teacher implementation of behavioral classroom management interventions, with those who do not receive this additional implementation support. We will measure teacher implementation outcomes (e.g., observed fidelity to behavioral classroom interventions) and student clinical outcomes (e.g., ADHD-related impairment, ADHD symptoms, student–teacher relationship, academic performance) before and after an 8-week intervention period for both groups; we will also measure teacher-reported acceptability, appropriateness, and feasibility for the PBMIR group following the intervention period.

**Discussion:**

If there is preliminary evidence of feasibility and effectiveness, this pilot study will provide the foundation for evaluation the PBMIR at a larger scale and the potential to improve outcomes for students with or at risk for ADHD.

**Trial registration:**

This clinical trial was registered at ClinicalTrials.gov. (https://clinicaltrials.gov/) on 8/5/2022 which was prior to the time of first participant enrollment. The registration number is: NCT05489081.

## Background

Behavioral classroom management interventions, delivered by teachers in classrooms, show evidence for improving student behavioral and academic engagement outcomes [4]. Evidence-based classroom management interventions emphasize both antecedent- and positive consequence-based approaches to manage student classroom behavior, and are consistent with schoolwide frameworks such as Positive Behavior Interventions and Supports (PBIS; [32, 33]. They can be delivered to all students in the class (i.e., universal or Tier 1 practices), as well as with students who need additional support (i.e., targeted or Tier 2 interventions). These interventions are important for supporting children with or at-risk for attention-deficit/hyperactivity disorder (ADHD), for whom they show strong evidence of success [9, 10]. However, teachers often do not use Tier 1 and Tier 2 behavioral classroom interventions as designed, if they use them at all [14, 23, 28]. This suggests a need for implementation strategies to support teachers in using these interventions. Implementation strategies are most likely to be effective when they are theory driven and target specific, malleable factors that influence provider behavior [22].

Behavioral classroom management interventions encompass a wide range of practices, which can vary along several dimensions, including whether they are designed to be applied to the entire class (i.e., Tier 1), or to targeted students only (i.e., Tier 2); whether they use antecedents, consequences, or both; and the time and effort they require to implement. Evidence suggests that teachers use some specific interventions more than others [28] and our prior work indicates that teachers’ intentions to use behavioral classroom interventions vary by specific intervention [20]. Our prior work also found that teacher-reported barriers and facilitators to using behavioral interventions differ between Tier 1 and Tier 2 interventions [21]. Together, this suggests that implementation strategies to support teachers in using behavioral classroom interventions should be tailored to specific intervention components.

The Theory of Planned Behavior [1] may be useful for developing implementation strategies because it delineates two sets of factors that may influence teachers’ implementation: factors that promote their intentions to implement an intervention and factors that promote their ability to act on strong intentions [13]. It also delineates specific psychological determinants of intentions: attitudes (e.g., whether a teacher “likes” or “dislikes” behavioral classroom management strategies for ADHD), social norms (e.g., whether the teacher perceives that other teachers use behavioral classroom management strategies or whether their supervisor expects them to use them), and self-efficacy (e.g., whether teachers believe that they have the necessary skills to successfully implement behavioral classroom management practices). These determinants provide potential mechanisms for increasing implementation among teachers who report low intentions to use a behavioral intervention [13]. At the same time, for teachers who do not implement a behavioral intervention despite positive intentions to do so, implementation strategies should target teachers’ ability to act on their intentions [13]. Theories of habit formation [25], which propose that automatic behavior is triggered by situational or contextual cues, suggest specific strategies, such as reminders in the environment, to use when intentions are strong but not acted upon. For example, if a teacher is ambivalent about using a behavioral classroom intervention, they may benefit from messages targeting attitudes or norms (e.g., narrative from other teachers about how they have found it helpful). On the other hand, if a teacher intends to use the intervention but struggles to actually do so within a busy classroom environment, they may benefit from reminders or strengthening habits.

Our team developed an implementation resource package (“Positive Behavior Management Implementation Resources, PBMIR”) to support teachers in using Tier 1 and Tier 2 behavioral classroom interventions, particularly with students with elevated levels of hyperactivity, inattention or impulsivity. The resource package is informed by the Theory of Planned Behavior [1] and theories of habit formation [25], to target attitudes, norms, self-efficacy, habits, and reminders; it specifically targets barriers and facilitators identified by teachers as important in a previous study [21]. It uses a modular format to support teachers in using four key behavioral classroom management interventions: behavior-specific praise (i.e., providing frequent verbal acknowledgment by specifically labeling praise-worthy behavior; e.g., [6], precorrections (i.e., reminding students about behavioral norms prior to a time when behaviors of concern might be likely; e.g., [7]; brief and specific behavior corrections (i.e., consistently correcting behavior in a clear, concise and calm way; e.g., [28]; and use of daily behavior reports to provide feedback on specific behavioral goals (e.g., [28]. The PBMIR also includes two additional modules to support factors that were identified as critical for implementation of the target interventions: student–teacher relationships and adult wellness.

We developed the PBMIR through an iterative, community-partnered process, in which we made revisions based on feedback obtained from teachers during a series of try-outs of versions of the PBMIR; we interpreted try-out data and made revisions in partnership with a Program Development Team of stakeholders (e.g., teachers, coaches, administrators). We designed this resource package to fit within existing school structures, align with school-wide Positive Behavior Interventions and Supports (PBIS), and be feasible to sustain with existing resources. If effective, deploying this resource package as an implementation strategy at scale may help address the critical need of supporting teachers in using evidence-based behavioral classroom interventions. An important first step, though, is to conduct a pilot study testing the feasibility, acceptability, and preliminary effectiveness of the PBMIR.

### Aims

The main objective of this study is to pilot test the implementation strategy resource package. We will evaluate the teacher-reported feasibility, acceptability and appropriateness of the resource package. We will also collect data on teacher implementation outcomes and child effectiveness outcomes to examine the promise of the resource package.

## Method

### Setting and sample

The study will be conducted in elementary or elementary-middle schools from a large urban school district in the Northeast United States. The district’s student body is racially and ethnically diverse (about 50% Black/African American, 21% Hispanic/Latino, 14% Non-Hispanic White, 7% Asian, and 5% Multiracial or Other races). Approximately 80% of these students live in households that are income-eligible for free or reduced-price meals. We will recruit schools that have already adopted schoolwide Positive Behavioral Interventions and Supports (PBIS) and will obtain written permission from principals for their schools’ participation, following school district processes.

Our target sample size is 30 teachers (teaching grades Kindergarten through 5th grade; with students of ages approximately 5 through 12) within participating schools over a two-year period. Teachers will nominate students in their classroom to participate (see “Procedures” section). We will enroll 2 students per teacher, for a target sample size of 60 students. These sample sizes were selected to be consistent with guidelines for pilot studies to determine the feasibility of recruiting participants, collecting outcome data, and retaining participants in the protocol [34].

### Procedures

#### Recruitment and informed consent

All procedures are approved by the institutional research board at the Children’s Hospital of Philadelphia and the research review committee of the participating school district. Any protocol amendments will be reviewed and approved by the IRB and school district review board prior to any changes taking place, and ClinicalTrials.gov will be updated to reflect any relevant changes.

Teachers will be recruited from participating schools. See Table 1 for the schedule of enrollment, interventions, randomization, and data collection. After providing informed consent for their participation, teachers will nominate two students from their classroom to participate. Teachers will be instructed to nominate students who “show elevated levels of inattentive, hyperactive or impulsive behaviors;” whom “you think may benefit from increased use of positive behavior management practices,” and “for whom you could use additional support in managing their behaviors.” Teachers will complete the Impairment Rating Scale (IRS; [11], a 7-point scale ranging from 0 to 6, for each of these students, without providing identifying information about the student. To meet inclusion criteria, a student must receive a score of 3 or greater on at least one IRS domain. If one or more of the nominated students does not meet this criterion, the teacher will nominate an additional student until two students are identified who meet this criterion. Students will be excluded from participation if they have a special education classification of intellectual disability, if their primary presenting concern is psychosis or autism spectrum disorder based on parent- or teacher- report, or if they present as in acute risk of harm to self or others, such that participation would be clinically inappropriate because they warrant more intensive intervention.

**Table 1:**
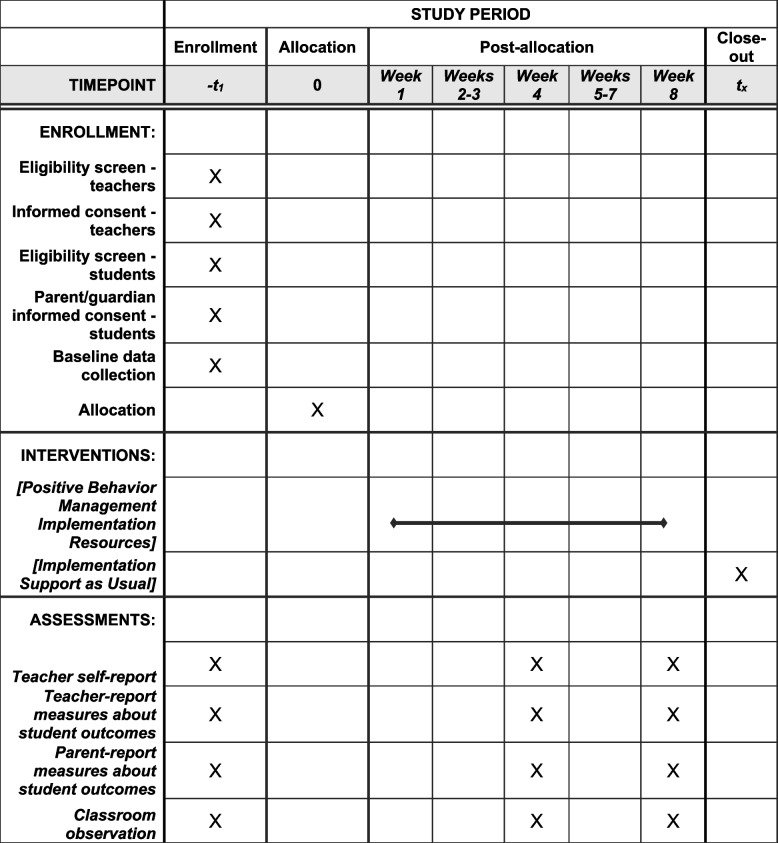
Schedule of enrollment, interventions, and assessments

For each student who meets criteria, teachers will be asked to contact the student’s parent/legal guardian to obtain verbal permission for the research team to contact them, and for the teacher to provide the research team with their contact information. If a student’s parent/legal guardian declines permission or cannot be reached, the teacher will nominate an additional student and will complete an IRS screening form regarding the student. If it is not possible to identify two children within a classroom who meet screening criteria and for whom informed consent can be obtained, we will allow the teacher to participate with one enrolled child.

After permission from the parent/legal guardian is obtained, the teacher will provide the research team with the student’s name, parent’s name, contact information, and preferred method of contact. The research team will reach out to the parent/legal guardian and obtain their informed consent for the student’s participation.

#### Randomization

Teachers will be randomized to condition blocked on grade level (i.e., grades K-2 vs. 3–5) and school. The principal investigator will generate the allocation sequence using online software (Sealed Envelope Ltd., 2022.) Students will be assigned to their teachers’ condition. A member of the research team will notify teachers of their assigned condition after baseline data collection is complete. If they are assigned to the “PBMIR” condition, the research team will schedule a meeting with them the week following randomization to provide them with a copy of the PBMIR, and the 8-week time period will start at that meeting. If they are assigned to the “Implementation Support as Usual” condition, the 8-week time period will start the following week, in order to ensure the timelines are comparable between conditions. Teachers in the “Implementation Support as Usual” condition will be permitted to receive any implementation support that they would otherwise receive related to classroom management (which could include trainings or instructional coaching), and this support will be documented via teacher report at endpoint. Teachers in this condition will receive a copy of the PBMIR electronic, written, and tangible resources following post- data collection to thank them for their participation. Children in both conditions will continue to receive any therapy or medication that they would otherwise receive during the study period, and parents will be asked to report about any medication their child takes at baseline and endpoint.

#### Positive Behavior Management Implementation Resources

The Positive Behavior Management Implementation Resources (PBMIR), previously developed using an iterative, community-partnered process, is organized into six modules: (1) student–teacher relationships; (2) adult wellness; (3) behavior-specific praise; (4) precorrections; (5) calm behavior-specific corrections; and (6) daily behavior reports.

Teachers will engage with the resource package for a period of 8 weeks. Each teacher is assigned a guide, who consults with the teacher during four, brief (i.e., 15–20 min) meetings during this period to support their engagement. The guide assists the teacher with goal setting, identifying relevant resources, building motivation, problem solving, and reflecting on progress. The guide supports the teacher in selecting modules to work on, working within a consistent structure: first focusing on student–teacher relationships (either class-wide or with the specific focal student), then focusing on behavior-specific praise, then proceeding to either precorrections or calmly-delivered behavior-specific corrections, and moving on to daily behavior reports last. Adult wellness resources for teachers are used if and when relevant.

Guides are supported by a written manual that consists of four sections: (1) an introduction with an overview, guiding beliefs (e.g., teachers are experts in their classrooms and their professional practice), and policies (e.g., confidentiality, scheduling); (2) an overview of the Theory of Planned Behavior; (3) process strategies (i.e., rapport building, motivational interviewing, goal setting, problem solving), and session agendas (i.e., a broad outline for the first session, follow-up sessions, and final session). Individuals serving in the guide role should be familiar with the elementary school classroom context, child development, and core behavioral principles, but guides do not need to have a specific educational background.

In addition to guide meetings, the resource package includes the following components: (1) written one-page documents (e.g., overview, tip sheet with messages targeting attitudes, norms, and strategies to use reminders, document with sentence stems to provide sample language); (2) fillable planning guides; (3) optional text message reminders, for which teachers can select the frequency and time to receive them; (4) the option to follow an ‘Instagram’ account with reminder posts and tips; (5) videos about the target practices, including teacher testimonials; (6) a notepad to use for self-monitoring; and (7) tangible resources and reminders, including stickers and sticky notes. Teachers set their goals and choose the resources to use in collaboration with their guide.

## Measures

### Feasibility of research procedures

#### Recruitment rate

We will track the number of teachers and students enrolled in the study. We will also examine the proportion of nominated and eligible students for whom informed consent is obtained.

#### Response rate

We will calculate the proportion of parent- and teacher- report surveys completed at each time point out of the number of teachers and students enrolled and randomized across conditions.

#### Retention rate

We will record the number of teachers and students who withdraw from the study and will compute the retention rate for each group of participants (i.e., teachers, students) as the proportion of participants who do not withdraw out of the number of enrolled and randomized teachers and children across conditions.

#### Engagement with the resource package

We will track teachers’ use of specific aspects of the resource package by tracking: 1) the number of guide meetings attended by each teacher in the PBMIR condition; 2) the percentage of teachers in the PBMIR condition who agree to receive text message reminders; and 3) the percentage of teachers in the PBMIR condition who choose to follow the Instagram account.

### Teacher implementation outcomes

#### Acceptability

Teachers in the resource package condition will complete the Acceptability of Intervention Measure (AIM; [37], which consists of four items (e.g., “[Intervention] is appealing to me”) on a 5-point Likert scale, regarding the resource package. Teachers will complete this measure at the end point, following their 8-week participation period. This measure has shown acceptable reliability (alpha $\ge$ 0.83) and test–retest reliability (Pearson correlations above 0.70) in prior samples.

#### Appropriateness

Teachers will complete the Intervention Appropriateness Measure (IAM; [37], which consists of four items (e.g., “[Intervention] seems suitable”) on a 5-point Likert scale, regarding the resource package. Teachers will complete this measure at the end point. This measure has shown acceptable reliability (alpha $\ge$ 0.87) and test–retest reliability (Pearson correlations above 0.70) in prior samples.

#### Feasibility

Teachers will complete the Feasibility of Intervention Measure (FIM; [37], which consists of four items (e.g., [Intervention] seems easy to use”) on a 5-point Likert scale, regarding the four target interventions supported by the resource package. Teachers will complete this measure at the end point. This measure has shown acceptable reliability (alpha $\ge$ 0.88) and test–retest reliability (Pearson correlations above 0.70) in prior samples.

#### Fidelity

A member of the study team will conduct classroom observations at the baseline, midpoint and end point of study participation for teachers in both conditions. These observations will use a modified version of the Student Behavior Teacher Response (SBTR) scale [27]. Observers will record counts of focal student norm violations and teacher responses to norm violations (i.e., no response, inappropriate response, appropriate response). Observers will also record counts of teacher use of behavior-specific praise, unlabeled praise, precorrections (directed at the focal students and overall), and teacher behaviors related to using daily behavior reports for focal students. We will observe these practices at both the universal and targeted levels because three of the target practices can be employed class-wide. Finally, the observer will rate the teachers’ global competence regarding classroom management and supportive relationships (with the whole class and with each target student) on a 7-point scale (from “poor” to “excellent”) following each observation. Approximately 20% of observations will be conducted by two observers so that inter-observer agreement can be calculated.

### Child outcomes

#### Student–teacher relationship scale

Teachers will complete the Student–Teacher Relationship Scale [29] about participating children at the baseline and the end point. This scale assesses teachers’ perceptions of their relationship with individual students. The STRS has shown adequate test–retest reliability (r = 0.89) and good internal consistency (α = 0.89; [29]. It generates three subscales (conflicts, closeness, dependency) and an overall total score. The total score will be used as a secondary outcome measure.

#### ADHD symptoms and impairment

Teachers and parents will complete the NICHQ Vanderbilt Scale [38] about participating children at the baseline and the end point. The inattention and hyperactivity/impulsivity scales on the Vanderbilt have high internal consistency, acceptable test–retest reliability, and convergent validity [3]. Teacher-report and parent-report on items related to adaptive functioning (e.g., “disrupting class,” “relationship with peers”) will be used as a primary outcome measure, and teacher-reported and parent-reported inattention and hyperactivity/impulsivity symptoms will be used as secondary outcome measures.

#### Academic performance rating scale

Teachers will complete the Academic Performance Rating Scale [8] about participating children at the baseline and the end point. This scale assesses teacher judgment of students’ academic functioning across two subscales: Academic Success (i.e., academic achievement) and Academic Productivity (i.e., day-to-day performance). The subscales are each measured by 8 items, rated on 5-point scales. They have acceptable internal consistency (0.72–0.95), stability (0.88–0.95), criterion-related validity, and sensitivity to intervention [12, 24]. Teacher-reported Academic Productivity will be used as a primary outcome measure and teacher-reported Academic Success will be used as a secondary outcome measure.

#### Direct behavior rating multi-item scales (DBR-MIS)

Teachers will complete ratings of child behavior using Direct Behavior Rating Multi-Item Scales (DBR-MIS; see [36]. For this study, teachers will rate behaviors in two domains: Engagement (5 items rating frequency of these behaviors on a 7-point scale ranging from Never to Almost Always) and Disruptive Behavior (5 items rating degree to which each item is a problem on a 7-point scale ranging from Not a Problem to Serious Problem). These domains of the DBR-MIS have demonstrated treatment sensitivity [17]. Teachers will complete these ratings at baseline, midpoint and endpoint. The average rating within domains will be used as secondary outcome measures.

#### Homework Performance questionnaire–parent form (HPQ-P)

Parents will complete the Homework Performance Questionnaire-Parent Form [30] at baseline and endpoint. Parents will complete this measure for target students in grades 1–5. The student self-regulation factor, which has shown strong internal consistency (i.e., alpha's between 0.92 and 0.94; [30] will be used as a secondary outcome measure.

### Potential mediators

#### Teacher intentions questionnaire

Teacher intentions to implement each of the four intervention components will be measured using a standardized 4-item questionnaire [31]. The items will use validated stems designed to probe provider intentions to use a specific practice (e.g., “I intend to…”), measured on a 7-point scale from “Strongly disagree” to “Strongly agree.” Teachers will be asked to complete these items “regarding students in your class who show elevated levels of inattentive, impulsive, or hyperactive behaviors.” Additionally, teachers will be asked to complete these items regarding participating children after the children have enrolled in the study.

#### Teacher determinants of intentions questionnaire

Teachers will complete a questionnaire with validated, standardized item stems (Fishbein and Ajzen, 2010) to report on their attitudes, norms, and self-efficacy for using behavioral classroom management interventions “regarding students in your class who show elevated levels of inattentive, impulsive, or hyperactive behaviors.”

#### Teacher self-rated habit index (SRHI)

Teachers will complete the SRHI, a 12-item scale that assesses the automaticity of a behavior, as well as its frequency of repetition and assimilation into one’s self-identity, regarding their use of behavior-specific praise at baseline and end point [35]. The SRHI has shown high internal consistency (i.e., alpha’s between 0.85 and 0.95) and convergent validity (i.e., correlation of *r* = 0.58 with response-frequency measure; [35].

#### Barriers and facilitators questionnaire

Teachers will report on the extent to which each of 10 potential barriers (five classified as beliefs that weaken intentions, and five classified as challenges that interfere with execution) and 12 facilitators (three classified as beliefs that strengthen intentions, seven classified as factors that assist with executing, and two classified as factors that assist with learning how to use the practice) impact their use of behavior-specific praise at baseline and endpoint. We developed these items from qualitative interview data from teachers in an earlier study [21].

#### Analytic approach

The primary goals of the analyses are to examine the feasibility and preliminary effectiveness of the PBMIR package. We will examine the recruitment rate, response rate, retention rate to assess the feasibility of the research procedures, inform adjustments to procedures as necessary, and inform sample size estimates in a larger trial as indicated. Consistent with recommendations [2] we will use traffic light criteria to determine whether the research protocol should proceed to a full trial unchanged (“green”), with adaptations (“amber”), or not at all (“red”). The specific criteria are listed in Table 2.

**Table 2:** Traffic light criteria

	Green (proceed to full trial without changes to research protocol)	Amber (adapt the protocol prior to proceeding to full trial)	Red (not progress to large-scale RCT with current protocol)
Recruitment rate	Enroll at least 20 teachers and 40 students	Enroll at least 15 teachers and 30 students	Enroll less than 15 teachers or 30 students
Response rate	Average response rate ≥ 75% across time points and conditions	Average response rate 50–74% across time points and conditions	Average response rate ≤ 50% across time points and conditions
Retention rate	Retention ≥ 75% for teachers and students across conditions	Retention 50–74% for teachers and students across conditions	Retention ≤ 50% for teachers and students across conditions

We use descriptive statistics to examine teacher-reported acceptability, appropriateness, and feasibility of the implementation resource package. We then will conduct independent sample *t* tests to examine whether the intervention and control groups differ on baseline teacher and student outcomes.

To compare teacher use of the four behavioral classroom interventions between the implementation strategy and control group, we will estimate means, standard deviations (SD), and 95% confidence intervals (CI) of observed teacher use of the four interventions and teacher global competence ratings. Similarly, to compare child-level outcomes between the groups, we will estimate means, SDs, and 95% CI of child primary and secondary outcomes. We will examine baseline and post-intervention scores, as well as change scores. We will compute effect sizes for each outcome measure by calculating the difference in change scores for the implementation strategy versus control group between post intervention and baseline and dividing this amount by the pooled standard deviation of the change score for implementation strategy and control [26].

Although the study is not powered for significance testing, we will conduct exploratory analyses examining between-group differences. We will use multi-level linear models, implemented in HLM software, to account for the nested data structure (i.e., students nested within teachers, nested within schools). We also will examine change in the measure of teacher intentions and teacher-reported implementation barriers, and explore with regression analyses the extent to which these variables have the potential to mediate the effect of intervention on outcomes, consistent with the conceptual model for mediation [15]. These analyses will determine whether there is preliminary evidence to support changes in intentions and specific barriers as plausible mechanisms for the implementation strategy.

## Discussion

The goal of this pilot study is to examine the feasibility and promise of the package of PBMIR, designed to help teachers strengthen student–teacher relationships and implement evidence-based behavioral classroom interventions, particularly for students with or at-risk for ADHD. If the pilot study results indicate that the PBMIR is acceptable, feasible and promising, this pilot study will provide the foundation for evaluating the PBMIR at a larger scale. Therefore, this research has the potential to ultimately lead to improved teacher effectiveness and student outcomes. Because the research is being conducted in a large, urban school district that serves a predominantly marginalized and minoritized student body, and the PBMIR is designed to be appropriate, feasible, and sustainable to implement in this context, it also may be appropriate for other large, urban school districts. Given that children spend much of their time in the classroom setting, improving the implementation of evidence-based practices for children with or at-risk for ADHD in this context offers considerable promise for improving population-level mental health and promoting health equity.

There are several notable strengths to this project. The PBMIR were developed through an iterative, community-partnered process, which is key to ensuring contextual appropriateness and feasibility [5, 18]. The implementation resource supports target practices that align with school-wide PBIS as implemented in the school district and uses terminology consistent with school and district initiatives. The PBMIR is informed by theory by targeting key constructs that shape individual’s intentions (i.e., attitudes, norms, self-efficacy) and their ability to act on intentions (i.e., habits, reminders); it was also informed by prior quantitative and qualitative data from teachers [20, 21]). The theory-driven, data-driven, and community-partnered foundational work for this study improves the likelihood that the PBMIR will be effective, acceptable, contextually appropriate, and feasible. We will collect data regarding both teacher implementation outcomes and student outcomes, using multiple methods (rating scale, direct observations) and informants (i.e., parent report, teacher report). Finally, we anticipate that the study will generate knowledge about potential mechanisms for implementation strategies (e.g., habits) that may generalize beyond the context of behavioral classroom interventions in schools.

We acknowledge several potential limitations, as well as potential practical or operational issues. First, the study is designed as a pilot study to establish feasibility and promise of the PBMIR and to lay the foundation for a larger scale trial; it is not powered for significance testing. Because of this goal, we are collecting data on a large number of outcome measures, and therefore may be at risk for type I and type II errors. Second, there are considerable challenges to participant recruitment, retention, and the implementation of research procedures in historically under-resourced schools. Many of these challenges have been heightened during the COVID-19 pandemic. We therefore anticipate challenges with recruitment and data collection, and are planning approaches to minimize burden, promote engagement using incentives ethically, and ensure alignment with school and district priorities. We also note that we do not plan to collect child-report outcome measures, which could add valuable information. Finally, we acknowledge that the PBMIR is designed as an individual, teacher-level implementation strategy, and does not target important school-level factors such as leadership and climate. School-level variability in these organizational factors may be important for the implementation and outcomes of the PBMIR, but the current study is not designed to examine these relationships, given the relatively small number of included schools.

If the pilot study results support the resource packages’ promise, the next step in this program of research would be to evaluate the PBMIR at a larger scale through an adequately-powered, randomized hybrid trial (i.e., a trial measuring both implementation outcomes and effectiveness outcomes simultaneously, see [19]. This would enable us to more definitively assess the PBMIR’s effectiveness regarding teacher implementation outcomes and student outcomes. Moreover, a larger scale trial would also support the examination of individual and organizational factors that serve as mediators and moderators of implementation outcomes.

## Data Availability

De-identified data will be available upon request from the corresponding author.

## Abbreviations

ADHD: Attention-Deficit/Hyperactivity Disorder
PBIS: Positive Behavior Interventions and Supports
PBMIR: Positive Behavior Management Implementation Resources
SBTR: Student Behavior Teacher Response
